# Disparities in Radiation Therapy Utilization for Solitary Plasmacytoma of Bone: A Surveillance, Epidemiology, and End Results Database Analysis

**DOI:** 10.3390/cancers17203294

**Published:** 2025-10-11

**Authors:** Kate Woods, Mitchell Taylor, Omar Hamadi, Aditya Sharma, Xudong Li, Peter Silberstein

**Affiliations:** 1 School of Medicine, Creighton University, Omaha, NE 68178, USA; 2 Department of Internal Medicine, Advocate Illinois Masonic Medical Center, Chicago, IL 60657, USA; mitchelltaylor.research@gmail.com (M.T.); dr.omarhamadi@hotmail.com (O.H.); 3 Department of Hematology and Oncology, Geisel School of Medicine at Dartmouth, Lebanon, NH 03755, USA; adityasharma96@hotmail.com; 4 Department of Orthopaedic Surgery, University of Virginia School of Medicine, Charlottesville, VA 22903, USA; xl2n@uvahealth.org; 5 Department of Internal Medicine, Division of Hematology and Oncology, Creighton University Medical Center, Omaha, NE 68124, USA; petersilberstein@creighton.edu

**Keywords:** solitary plasmacytoma of bone, SEER database, healthcare disparities

## Abstract

**Background/Objectives**: Solitary plasmacytoma of bone (SPB) results from abnormal proliferation of plasma cells and accounts for 2–5% of all plasmacytic malignancies. Radiation therapy is the standard of care in treating SPB due to its efficacy in controlling disease progression and optimizing patient survival. However, prior studies have highlighted disparities in radiation therapy receipt among various cancer types. In this study, we aim to investigate whether similar sociodemographic and clinical disparities exist in the treatment of SPB through use of the Surveillance, Epidemiology, and End Results (SEER) database. **Methods**: The SEER database was queried for biopsy-confirmed cases of SPB between 2000 and 2021 using the ICD-O-3 histology code 9731/3 and primary site codes C40.0–41.9. Chi-square tests, Fisher’s exact tests, and multivariable logistic regression were completed using SPSS v29.0.2, with significance set to *p* < 0.05. **Results**: A total of 4139 patients were identified, of which 75.3% received treatment with radiation therapy. Multivariable analysis revealed that low-income patients making less than $74,999 annually (aOR 0.80, 95% CI 0.67–0.97), as well as those from non-Hispanic Asian/Pacific Islander (aOR 0.49, 95% CI 0.33–0.73) and Hispanic (aOR 0.77, 95% CI 0.60–0.98) racial and ethnic groups, were significantly less likely to receive radiation therapy. **Conclusions**: These findings reveal notable disparities in radiation therapy utilization for SPB patients based on income and race and ethnicity, emphasizing the need for interventions to address systemic inequities, improve access to care, and ensure that all patients receive high-quality cancer care to optimize long-term outcomes.

## Simple Summary

Solitary plasmacytoma of bone (SPB) is a rare cancer treated most effectively with radiation therapy, which helps control disease progression and improve patient survival. However, in other cancers, prior research has shown that patients from certain racial and ethnic backgrounds are less likely to receive this treatment. This study explores whether similar disparities exist for SPB by analyzing data from thousands of patients across the U.S. Our aim is to identify if certain groups are less likely to receive recommended radiation therapy and to raise awareness for potential inequalities in cancer care. These findings could help guide future efforts to ensure fair treatment for all patients, regardless of background.

## 1. Introduction

Solitary plasmacytoma of bone (SPB) is a rare plasma cell dyscrasia, representing approximately 2–5% of all plasmacytic malignancies, and is distinguished by the presence of a single osteolytic bone lesion in the absence of systemic involvement [1]. SPB most frequently arises in the axial skeleton, particularly within the vertebrae, ribs, skull, and pelvis, leading to symptoms such as pathological fractures or spinal cord compression [2]. It also predominantly affects men, with 65% of patients being male [3]. The incidence of disease progression is high in SPB, with around two-thirds of cases eventually progressing to multiple myeloma within a decade [3,4]. Risk factors associated with progression are older age, tumor size > 5 cm, and the presence of high-risk cytogenic abnormalities [3,4]. Therefore, proper diagnosis and treatment of SPB are essential to mitigate progression to multiple myeloma.

Radiation therapy (RT) is considered the gold standard treatment for SPB due to its high efficacy in targeting clonal plasma cells while minimizing damage to surrounding tissue [5,6]. With response rates exceeding 80% [7], RT provides excellent local disease control, with a typical dose ranging from 35 to 50 Gy [8,9]. This treatment approach has proven to be highly effective in controlling disease progression and improving patient survival, particularly when utilized as a first-line therapy. Although surgery may be considered in cases of fractures or spinal instability [10], RT remains the standard of care, offering long-term disease control for a majority of SPB patients.

Despite the well-established efficacy of RT in treating SPB and other malignancies, disparities in its utilization have been observed across various cancer subtypes [11,12]. For example, in breast cancer, Black women are significantly less likely to receive adjuvant RT after breast-conserving surgery than White women (*p* < 0.001), contributing to poorer local control and survival outcomes [13]. Similarly, in prostate cancer, racial and ethnic minority groups are less likely to receive high-quality or guideline-concordant RT, including image-guided and dose-escalated techniques, compared to their White counterparts [12]. Factors such as sociodemographic characteristics, geographic location, and healthcare accessibility have been shown to influence whether patients receive RT, leading to significant variations in treatment across different populations and subsequent outcomes [11,12,13,14].

These established patterns of inequitable RT utilization in other malignancies motivated our investigation to determine whether similar disparities exist in the treatment of SPB. Although SPB is a rare plasma cell neoplasm, it shares with these other cancers a clear, evidence-based recommendation for definitive RT as the standard of care in SPB. Given the parallels in treatment modalities and the healthcare system through which oncologic care is delivered, it is reasonable to hypothesize that similar disparities may exist in SPB. By utilizing the Surveillance, Epidemiology, and End Results (SEER) database, our study aims to identify patterns in RT utilization for SPB, with a focus on sociodemographic and clinical factors that may contribute to unequal access to care. Understanding these disparities is crucial for addressing gaps in treatment delivery and ensuring that all patients, regardless of background, receive the optimal care necessary to improve their long-term outcomes. Moreover, identifying potential disparities in a rare cancer like SPB is important as they may go unrecognized in areas with less robust clinical infrastructure, such as underserved settings.

## 2. Materials and Methods

**Figure 1:**
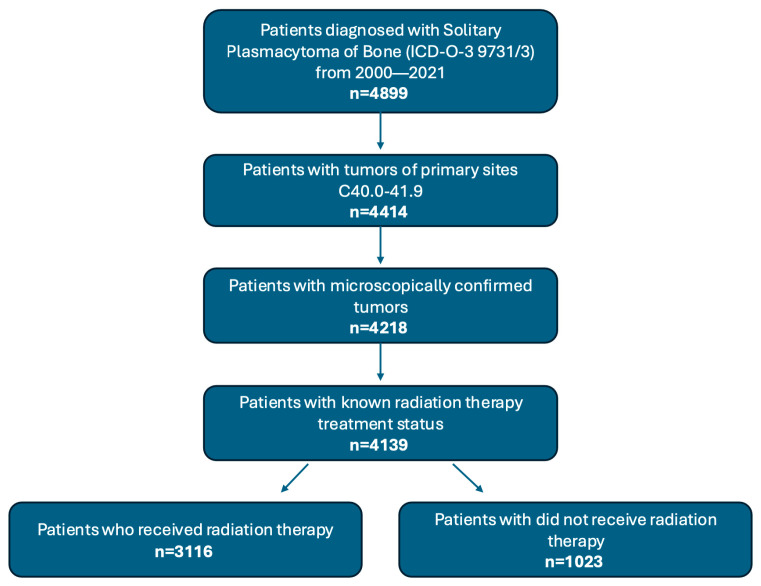
Flowchart of patient cohort identification and selection from the SEER database.

Patient data from the years 2000 to 2021 were obtained using SEER*Stat 8.4.4 (NCI/NIH, Bethesda, MD, USA). The SEER database compiles data from 17 cancer registries across the United States, covering about 26.5% of the U.S. population. Cases of SPB with histologic confirmation were identified using the International Classification of Diseases for Oncology 3rd edition codes 9731/3 and primary site codes C40.0–41.9 (Figure 1). Collected demographic data included patient age at diagnosis, sex, race and ethnicity, annual income, and residential classification as urban or rural. Rural-urban classification was determined based on SEER Rural-Urban Continuum codes established by the U.S. Department of Agriculture’s Economic Research Service. Annual income and rural–urban classification represent area-level variables assigned based on the patient’s ZIP code or census tract and were used as proxies for individual socioeconomic status and geographic access to care. Disease characteristics, including primary tumor location and disease stage, were recorded. The SEER historical staging framework categorizes malignancies into localized (confined to the organ of origin), regional (spread to nearby tissues or lymph nodes), and distant (metastasized to distant locations through direct extension, lymphatic spread, or non-contiguous dissemination). This system ensures compatibility across various staging methodologies used in different time periods. Collected treatment data included surgical intervention, chemotherapy, and RT. Only patients with documented RT treatment or no treatment were included.

Statistical analysis was performed using SPSS for Mac, version 29.0.2 (IBM Corp, Armonk, NY, USA). Chi-square tests and Fisher’s exact tests were used to assess associations between categorical variables and RT utilization. To identify factors independently associated with receipt of RT, a multivariable logistic regression model adjusting for age at diagnosis, sex, race and ethnicity, annual income, rural-urban living, primary tumor location, disease stage, surgical intervention, and chemotherapy was applied. Multicollinearity among covariates was assessed by calculating the variance inflation factors (VIFs) for the predictor variables included in the multivariable logistic regression model. All covariates demonstrated a VIF of less than 2, indicating that multicollinearity had a minimal effect on the model and that the regression estimates were not significantly distorted by highly correlated predictors. A *p*-value of <0.05 was considered statistically significant.

## 3. Results

A total of 4139 patients diagnosed with SPB were identified (Table 1). The majority of patients were male (61.6%) and identified as non-Hispanic (NH) White (66.0%), followed by Hispanic (of any race) (15.4%), NH Black (13.6%), NH Asian/Pacific Islander (API) (4.3%), and NH American Indian/Alaska Native (AIAN) (0.7%). The most common age group at diagnosis was 60–69 years, representing 26.8% of the cohort, followed by 70–79 years (22.4%) and 50–59 years (20.3%). The majority of patients resided in urban areas (86.5%), with only 13.5% living in rural settings. Socioeconomic data revealed that 51.5% of patients had an estimated annual income of less than $74,999, while 48.5% had an income of $75,000 or more. Regarding disease characteristics, the spine was the most common primary tumor location (45.2%), followed by the thoracic (16.0%), pelvic (16.0%), limbs (14.3%), and cranial bones (8.5%). The majority of patients were also diagnosed with localized disease stage (85.5%) compared to regional and distant disease stages (14.5%). Treatment data highlighted that RT was the most frequently utilized treatment modality, administered to 75.3% of patients, while chemotherapy was received by only 19.9% of the cohort. Surgical excision was performed in 25.0% of cases.

When comparing SPB patients who received RT to those who did not, RT was significantly associated with several clinicopathologic variables (Table 2). Age at diagnosis was significantly associated with RT receipt (*p* < 0.001), with younger patients more likely to receive RT compared to older patients. Annual income also showed a significant association (*p* = 0.005), where a higher proportion of patients with an income of <$74,999 received RT (50.2%) compared to those with incomes of $75,000+ (49.8%). Primary tumor location was significantly associated with RT receipt (*p* < 0.001), with a higher percentage of spinal tumors in the RT group (48.2% vs. 35.0%) and a higher percentage of thoracic tumors in the non-RT group (22.6% vs. 14.1%). Disease stage was also significantly associated with RT receipt (*p* < 0.001), where a higher percentage of patients with localized disease were treated with RT (88.2%) compared to patients with regional or distant disease stages (11.8%). No significant associations were observed between RT utilization and sex (*p* = 0.159), race and ethnicity (*p* = 0.053), rural-urban living (*p* = 0.874), or surgical excision (*p* = 0.143).

Multivariable logistic regression adjusting for age at diagnosis, sex, race and ethnicity, annual income, rural-urban living, primary tumor location, disease stage, surgical excision, and chemotherapy revealed that several demographic and clinicopathologic features were independently associated with RT receipt (Table 3). Age at diagnosis was significantly associated with patients aged 70–79 (adjusted odds ratio [aOR] 0.58, 95% confidence interval [CI] 0.36–0.93) and 80+ (aOR 0.34, 95% CI 0.21–0.55) exhibiting lower odds of receiving RT compared to those under 40 years of age. Race and ethnicity were also independently associated with RT receipt, where NH API (aOR 0.49, 95% CI 0.33–0.73) and Hispanic patients (aOR 0.77, 95% CI 0.60–0.98) were at reduced odds of receiving RT when compared to NH White individuals. Individuals with an annual income of <$74,999 were also less likely to receive treatment with RT (aOR 0.80, 95% CI 0.67–0.97) compared to those making $75,000+ annually. Primary tumor location also impacted RT utilization, with cranial (aOR 0.69, 95% CI 0.48–0.99) and thoracic tumors (aOR 0.60, 95% CI 0.45–0.82) being at lower odds of RT receipt, whereas spinal tumors were associated with higher odds of receiving RT (aOR 1.34, 95% CI 1.02–1.76). Patients who underwent surgical excision (aOR 1.25; 95% CI 1.01–1.55) and no chemotherapy (aOR 1.41; 95% CI 1.14–1.74) were also more likely to have been treated with RT.

## 4. Discussion

In this nationally representative cohort analysis of the SEER database, we highlight significant disparities in RT utilization for patients diagnosed with SPB, persisting even after adjusting for key covariates. Our study revealed that NH API and Hispanic patients diagnosed with SPB were at significantly lower odds of receiving RT, with NH API patients being 51% less likely and Hispanic patients being 23% less likely to undergo RT compared to their NH White counterparts. Additionally, income was significantly associated with RT utilization, as patients who earned less than $74,999 annually were 20% less likely to receive RT compared to patients who earned over $75,000. Other covariates found to be independently associated with RT utilization included age, primary tumor location, surgical excision, and chemotherapy use. The substantial disparities observed in our study, particularly in the context of race and ethnicity and income, raise significant concerns, given that the administration of RT in patients with SPB is associated with improved local control and progression-free survival and is considered the standard of care [5,6].

Consistent with our findings in an SPB cohort, other studies have similarly documented existing racial and ethnic disparities in RT utilization across various types of malignancies [13,15,16,17]. In a study by Smith et al. investigating RT utilization patterns following breast-conserving surgery (BCS) for breast cancer, significant racial and ethnic disparities were observed [13]. Their study of 34,080 breast cancer patients found that only 65% of Black women and 66% of women from other racial groups received RT after BCS, compared to 74% of White women (*p* < 0.001). Further multivariable analysis revealed that White women were more likely to receive RT than Black women (aOR 1.48; 95% CI 1.34–1.63) and women from other racial groups (aOR 1.22; 95% CI 1.04–1.42).

Another study by Lee et al. examining patterns of RT receipt in the care of prostate cancer patients found that Black males and those from other minority racial groups were less likely to receive external beam radiation therapy (EBRT) compared to White men [15]. In their study of 926 patients, 77% of White males were reported to have received EBRT that met all quality standards, while only 64% of Black patients and 62% of men from other racial and ethnic backgrounds received EBRT adhering to recommended guidelines (*p* < 0.01). Hispanic men and those from other racial groups (73%) were also less likely to receive image-guided radiation therapy compared to White (87%) or Black males (88%; *p* = 0.02). Additional multivariable analyses examining compliance with quality measures among patients who received EBRT further highlighted that Black patients and men from other minority racial groups experienced 46% (aOR 0.54; 95% CI, 0.32–0.89) and 51% (aOR 0.49; 95% CI, 0.27–0.91) reduced odds, respectively, compared to their White counterparts. These findings, in corroboration with those observed in our study of SPB patients, emphasize ongoing racial and ethnic disparities in RT utilization and adherence to quality standards across different malignancies.

To help eliminate disparities in RT utilization among patients with SPB, multifaceted interventions are needed. Medical literature robustly supports RT as the standard of care for SPB, a position reinforced by national guidelines from the National Comprehensive Cancer Network (NCCN) [5,18,19,20,21,22,23]. Despite these recommendations, however, our study suggests that adherence to these guidelines remains inconsistent for certain underserved groups. This gap between guideline recommendations and clinical practice may reflect systemic barriers, including unequal access to radiation facilities. Prior studies have demonstrated that Hispanic patients experience greater geographic barriers and travel distances to RT than their NH White counterparts, with Hispanic patients in rural and urban settings both affected [24]. This has important implications for outcomes for Hispanic cancer patients, as one study demonstrated that Hispanic women with breast cancer had lower odds of receiving treatment as the distance to the nearest radiation provider increased [25]. Interestingly, while our study noted reduced RT receipt in API patients, this may not necessarily reflect challenges in geographic access to radiation services, as our study noted no significant association between rural-urban residence and RT receipt (aOR 1.05; 95% CI 0.80–1.36). A study by Shrestha et al. noted that, while lack of transportation and time is a common healthcare barrier in Asian American populations, they found no association between geographic access to radiation oncologists and RT in API patients with non-small cell lung cancer [26]. Rather, the authors postulate that difficulties in taking time off work for repeated RT sessions might instead produce larger obstacles to RT receipt as opposed to geographic challenges [26]. To improve access to RT, health systems should focus on improving access to RT services in underserved communities, potentially through telemedicine consultations and/or mobile RT units. While still a relatively novel concept, semi-mobile radiation oncology units have been employed in various locations across the U.S. to provide treatment for smaller radiation oncology clinics [27]. A simulation analysis run by Price et al. demonstrated techno-economic feasibility of a fully mobile RT unit employed in rural Missouri [27]. Such technologies, if properly adapted and employed for real-world use, could significantly improve access to oncologic care in rural and underserved communities.

Our observed disparities may also be related to patient-level factors such as mistrust of the healthcare system, which is particularly common among racial and ethnic minorities. Prior studies consistently find higher levels of medical mistrust among minority patients, particularly Black and Hispanic individuals, compared to their White counterparts [28,29,30]. This mistrust is strongly associated with experiences of discrimination in healthcare settings, perceived lack of respect, poor communication, and historical awareness of unethical research practices involving minorities [28,29,30]. These factors contribute to lower engagement with healthcare services and may influence our observed disparities in rates of RT receipt among racial and ethnic minorities. Proposed interventions to combat medical mistrust are cultural humility training, community outreach, emphasizing patient-centered care, and leveraging trusted community leaders to rebuild trust among minority populations [31,32]. In the context of SPB oncologic treatment, this may involve patient navigation programs that include culturally tailored education and support to help mitigate non-clinical barriers to RT. Such patient-centered programs may also be utilized to address other potential barriers to radiation care, such as low health literacy and socioeconomic status, which disproportionately affect racial and ethnic minority groups [33,34].

Regarding socioeconomic status, our study found that low income was independently associated with lower odds of receiving RT. Current trends in the literature note similar disparities in cancer treatment access and delivery. A population-based study of the CDC’s National Program of Cancer Registries by Kava et al. reported that patients living in counties within the bottom 25% (OR 0.80; 95% CI 0.78–0.81) and middle 50% (OR 0.87; 95% CI 0.86–0.88) of economic status had significantly lower odds of receiving at least one of four possible standard cancer treatments, including RT, chemotherapy, surgery, or immunotherapy [35]. Their analysis considered factors such as unemployment rate, per capita market income, and poverty rate within each county [35]. The underlying causes of these disparities are likely multifactorial and may be related to economic deprivation or insurance status, both of which are related to socioeconomic status and have been shown to negatively impact outcomes and access to oncologic care [35,36,37]. Additionally, geographical barriers, such as long travel distances and a lack of local treatment facilities, disproportionately affect low-income patients [38,39] and further reduce likelihoods of receiving RT [40]. Improving communication between oncology providers and patients may serve as one pathway to mitigate these disparities. Previous studies have shown that physicians serving low-income communities often face challenges in clearly explaining disease processes and treatment options, which can hinder patient understanding and engagement [41,42]. Notably, Lineback et al. found that patients experienced greater satisfaction and fewer care delays when supported by a longitudinal caregiver—such as a social worker, advanced practice provider, or care coordinator—who was available throughout treatment to address concerns and help navigate barriers [41]. Reducing socioeconomic disparities in cancer care will require approaches such as enhanced communication, targeted patient support, and broader efforts to improve access for vulnerable populations.

While this study provides valuable insights into racial, ethnic, and economic disparities in RT utilization among patients with SPB, several limitations should be considered. For one, the SEER database does not report detailed information on certain patient-level factors that may influence RT decision-making, such as physician preferences or patient comorbidities. Additionally, the SEER database does not provide information on the specific dosing of RT, which is recognized to play an important role in treatment outcomes. This lack of data on radiation dosage may limit the accuracy of our conclusions regarding adherence to quality standards. There is also the potential for missing or incomplete data in the SEER database, which may impact the accuracy and generalizability of our findings. Additionally, nearly all of the data within the SEER database regarding treatment sequencing remains undocumented or missing, and the binary nature of treatment variables prevents distinguishing between patients who received RT alone, surgery alone, or both. As such, we cannot definitively determine whether patients who did not receive RT instead underwent surgery or chemotherapy as definitive therapy. Fortunately, we are confident that inclusion of surgery and chemotherapy in our multivariable model effectively adjusts for these factors and reduces potential confounding; however, because the majority of treatment sequencing data are missing, this remains a limitation that may still introduce bias in interpreting disparities in RT utilization.

This study is also limited by the use of SEER*Stat, which provides access only to recoded and de-identified variables in the standard SEER research files, restricting the granularity of socioeconomic and treatment data and potentially limiting the depth of analysis. Our analysis also used standard logistic regression, which assumes independent observations. Because the SEER dataset lacks hierarchical identifiers, we could not account for clustering by region or registry, which may introduce residual correlation and limit the precision of some estimates. Future studies with identifiable clustering could apply multilevel modeling to address this limitation. Additionally, the observational nature of this study limits the ability to establish causality, and our study’s reliance on retrospective data may introduce biases due to incomplete documentation or variations in how information was recorded over time. Despite these limitations, the findings from this study highlight critical disparities and underscore the need for further research to better understand underlying factors contributing to inequities in cancer care. Future studies should aim to further identify and characterize the factors driving racial, ethnic, and economic disparities in RT utilization, including comorbidities and healthcare access. Additionally, while our study utilized the full SEER dataset to capture SPB treatment patterns across all adult age groups, future studies may consider using SEER-Medicare linked data to provide more detailed information on radiation dosing, chemotherapy protocols, and surgical procedures among patients aged 65 and older.

## 5. Conclusions

Solitary plasmacytoma of bone (SPB) is a rare plasma cell malignancy that is most often treated with radiation therapy due to its efficacy in targeting neoplastic cells, providing disease control and limiting progression, and improving patient outcomes. Our study reveals disparities in radiation therapy utilization for patients with SPB. Those who were low-income and from Asian/Pacific Islander and Hispanic racial and ethnic groups were significantly less likely to receive radiation therapy. These findings emphasize ongoing disparities in radiation therapy use, which are found across a variety of malignancies. Urgent intervention is necessary to address systemic inequalities impacting access to care for low-income and racial and ethnic minority groups, so that all patients may receive the highest standard of cancer care regardless of background.

## Figures and Tables

**Table 1:** Clinicopathological Features in a Solitary Plasmacytoma of Bone Cohort.

*n* = 4139	Frequency (*n*)	Percentage (%)
**Age at diagnosis (years)**		
<40	205	5.0
40–49	423	10.2
50–59	842	20.3
60–69	1109	26.8
70–79	929	22.4
80+	631	15.2
**Sex**		
Male	2549	61.6
Female	1590	38.4
**Race and ethnicity**		
NH White	2719	66.0
NH Black	560	13.6
NH API	177	4.3
NH AIAN	28	0.7
Hispanic (any race)	636	15.4
**Annual income ** **^∞^**		
<$74,999	2056	51.5
$75,000+	1940	48.5
**Rural-urban living ^σ^**		
Urban	3572	86.5
Rural	556	13.5
**Primary tumor location**		
Limbs	547	14.3
Cranial	325	8.5
Spine	1733	45.2
Thoracic	613	16.0
Pelvic	612	16.0
**Disease stage**		
Localized	3056	85.5
Regional & distant	517	14.5
**Surgical excision**		
Yes	1030	25.0
No	3093	75.0
**Chemotherapy**		
Yes	823	19.9
No	3316	80.1
**Radiation therapy**		
Yes	3116	75.3
No	1023	24.7

AIAN American Indian/Alaska Native; API Asian or Pacific Islander; NH, non-Hispanic; SEER, Surveillance Epidemiology and End Results. **^∞^** Annual income data obtained from United States Census Bureau 5-year estimates. **^σ^** Rural vs. urban living was determined using the SEER Rural-Urban Continuum codes initially defined by the Economic Research Service at the United States Department of Agriculture.

**Table 2:** Comparison of Clinicopathological Features by Receipt of Radiation Therapy in a Solitary Plasmacytoma of Bone Cohort.

Total *n* = 4139	RT (*n* = 3116)	No RT (*n* = 1023)	*p*-Value
**Age at diag** **nosis (years)**			**<0.001** °
<40	168 (5.4%)	37 (3.6%)	
40–49	334 (10.7%)	89 (8.7%)	
50–59	679 (21.8%)	163 (15.9%)	
60–69	848 (27.2%)	261 (25.5%)	
70–79	680 (21.8%)	249 (24.3%)	
80+	407 (13.1%)	224 (21.9%)	
**Sex**			0.159 ^♢^
Male	1938 (62.2%)	611 (59.7%)	
Female	1178 (37.8%)	412 (40.3%)	
**Race and ethnicity**			0.053 °
NH White	2080 (67.0%)	639 (63.0%)	
NH Black	421 (13.6%)	139 (13.7%)	
NH API	120 (3.9%)	57 (5.6%)	
NH AIAN	21 (0.7%)	7 (0.7%)	
Hispanic (any race)	464 (14.9%)	172 (17.0%)	
**Annual income ^∞^**			**0.005** ^♢^
<$74,999	1507 (50.2%)	549 (55.3%)	
$75,000+	1496 (49.8%)	444 (44.7%)	
**Rural-urban living ^σ^**			0.874 ^♢^
Rural	417 (13.4%)	139 (13.6%)	
Urban	2692 (86.6%)	880 (86.4%)	
**Primary tumor location**			**<0.001** °
Limbs	422 (14.2%)	125 (14.4%)	
Cranial	223 (7.5%)	102 (11.8%)	
Spine	1429 (48.2%)	304 (35.0%)	
Thoracic	417 (14.1%)	196 (22.6%)	
Pelvic	471 (15.9%)	141 (16.2%)	
**Disease stage**			**<0.001** ^♢^
Localized	2359 (88.2%)	697 (77.7%)	
Regional & distant	317 (11.8%)	200 (22.3%)	
Surgical excision *	2320 (74.5%)	773 (76.8%)	0.143 ^♢^
Chemotherapy *	578 (18.5%)	245 (23.9%)	**<0.001** ^♢^

Significant *p*-values (<0.05) are in bold. AIAN American Indian/Alaska Native; API Asian or Pacific Islander; NH, non-Hispanic; RT, radiation therapy; SEER, Surveillance Epidemiology and End Results. ° Test statistic calculated using chi-squared test. ^♢^ Test statistic calculated using two-sided Fisher’s exact test. * In comparison to not receiving this treatment. **^∞^** Annual income data obtained from United States Census Bureau 5-year estimates. **^σ^** Rural vs. urban living was determined using the SEER Rural-Urban Continuum codes initially defined by the Economic Research Service at the United States Department of Agriculture.

**Table 3:** Multivariable Analysis Identifying Factors Associated with Radiation Therapy Utilization.

Total *n* = 4139	aOR ‡	95% CI	*p*-Value
**Age at diagn** **osis (years)**			
<40	Reference		
40–49	0.75	0.45–1.26	0.281
50–59	0.97	0.59–1.57	0.885
60–69	0.68	0.43–1.10	0.113
70–79	0.58	0.36–0.93	**0.023**
80+	0.34	0.21–0.55	**<0.001**
**Sex**			
Male	Reference		
Female	0.98	0.82–1.17	0.800
**Race and ethnicity**			
NH White	Reference		
NH Black	0.80	0.62–1.03	0.087
NH API	0.49	0.33–0.73	**<0.001**
NH AIAN	0.97	0.26–3.63	0.969
Hispanic (any race)	0.77	0.60–0.98	**0.034**
**Annual income ^∞^**			
$75,000+	Reference		
<$74,999	0.80	0.67–0.97	**0.020**
**Rural-urban living ^σ^**			
Urban	Reference		
Rural	1.05	0.80–1.36	0.742
**Primary tumor location**			
Limbs	Reference		
Cranial	0.69	0.48–0.99	**0.045**
Spine	1.34	1.02–1.76	**0.033**
Thoracic	0.60	0.45–0.82	**0.001**
Pelvic	0.93	0.68–1.28	0.669
**Disease stage**			
Localized	Reference		
Regional & distant	0.55	0.43–0.71	**<0.001**
**Surgical excision**			
No	Reference		
Yes	1.25	1.01–1.55	**0.038**
**Chemotherapy**			
Yes	Reference		
No	1.41	1.14–1.74	**0.001**

NH, non-Hispanic; AIAN, American Indian/Alaska Native; aOR, adjusted odds ratio; API, Asian or Pacific Islander; CI, confidence interval; RT, radiation therapy. ‡ Adjusted odds ratio refers to odds of receipt of RT after adjusting for important covariates. Significant *p*-values (<0.05) are in bold. **^∞^** Annual income data obtained from United States Census Bureau 5-year estimates. **^σ^** Rural vs. urban living was determined using the SEER Rural-Urban Continuum codes initially defined by the Economic Research Service at the United States Department of Agriculture.

## Data Availability

The data utilized in this manuscript are available upon request from the corresponding author.

## Abbreviations

The following abbreviations are used in this manuscript:

SPB	Solitary plasmacytoma of bone
RT	Radiation therapy
SEER	Surveillance, Epidemiology, and End Results
AIAN	American Indian/Alaska Native
NH	non-Hispanic
API	Asian or Pacific Islander
aOR	Adjusted odds ratio
CI	Confidence interval
